# Wearable-based accelerometer activity profile as digital biomarker of inflammation, biological age, and mortality using hierarchical clustering analysis in NHANES 2011–2014

**DOI:** 10.1038/s41598-023-36062-y

**Published:** 2023-06-08

**Authors:** Jinjoo Shim, Elgar Fleisch, Filipe Barata

**Affiliations:** 1 Centre for Digital Health Interventions, Department of Management, Technology, and Economics, ETH Zurich, Zurich, Switzerland; 2 Centre for Digital Health Interventions, Institute of Technology Management, University of St. Gallen, St. Gallen, Switzerland

**Keywords:** Disease prevention, Biomarkers, Risk factors, Machine learning

## Abstract

Repeated disruptions in circadian rhythms are associated with implications for health outcomes and longevity. The utilization of wearable devices in quantifying circadian rhythm to elucidate its connection to longevity, through continuously collected data remains largely unstudied. In this work, we investigate a data-driven segmentation of the 24-h accelerometer activity profiles from wearables as a novel digital biomarker for longevity in 7,297 U.S. adults from the 2011–2014 National Health and Nutrition Examination Survey. Using hierarchical clustering, we identified five clusters and described them as follows: “High activity”, “Low activity”, “Mild circadian rhythm (CR) disruption”, “Severe CR disruption”, and “Very low activity”. Young adults with extreme CR disturbance are seemingly healthy with few comorbid conditions, but in fact associated with higher white blood cell, neutrophils, and lymphocyte counts (0.05–0.07 log-unit, all *p *< 0.05) and accelerated biological aging (1.42 years, *p *< 0.001). Older adults with CR disruption are significantly associated with increased systemic inflammation indexes (0.09–0.12 log-unit, all *p *< 0.05), biological aging advance (1.28 years, *p *= 0.021), and all-cause mortality risk (HR = 1.58, *p *= 0.042). Our findings highlight the importance of circadian alignment on longevity across all ages and suggest that data from wearable accelerometers can help in identifying at-risk populations and personalize treatments for healthier aging.

## Introduction

The widespread adoption of personal digital devices, such as smartphones and wearables, offers an unprecedented potential for data collection to assess human health and disease states. By passively and continuously measuring, in-built device sensors enable us to capture various essential health functions (e.g., skin temperature, sleep–wake cycles, and heart rate)^1^ and factors of the surrounding environment (e.g., light exposure)^2^ and lifestyle (e.g., physical activity and diet)^1,3^ in a real-world context over extended periods. These digitally captured physiological and behavioral measures, also known as digital biomarkers, explain, influence, or predict health-related outcomes^4^. Digital biomarkers can mirror a person’s daily living and behavioral patterns more accurately and objectively, and thus, may substitute or complement routine clinical evaluations or self-assessments^5^.

Recent research has proposed that digital biomarkers for longevity could be used to identify individuals at higher risk for age-related diseases and to monitor the effectiveness of interventions aimed at promoting healthy aging^6,7^. This is particularly relevant given the increasing burden of age-related diseases on healthcare systems and society^8^. Currently, measures of health and longevity are based on factors such as inflammation^9^, biological age^10^, and mortality^11^. While these predictors can provide a better understanding of an individual's life expectancy than chronological age, their potential for digitization has not been extensively studied^12,13^. A digital biomarker for longevity would not only provide a digital measure of lifespan, but also enable personalized interventions for healthy aging, such as nutritional and pharmacological interventions. This aligns with the concept of precision medicine, which emphasizes prediction, prevention, personalization, and participation over a one-size-fits-all approach^14^.

The circadian rhythm, an endogenous 24-h cycle regulated by the master clock in the suprachiasmatic nucleus of the brain, has also been recognized as a crucial factor for maintaining optimal health and healthspan^15^. The circadian rhythm regulates various physiological, biological, and behavioral processes in the body, including sleep–wake cycles, hormone production, metabolism, and immune function^16^. Although external time cues such as “zeitgeber” (24-h light–dark cycle) can influence the circadian rhythm, it is predominantly controlled by endogenous factors, which are deeply rooted in an individual's genetic makeup. Emerging evidence strongly suggests that the disturbance or misalignment of the circadian rhythms has profound implications for health outcomes, including disrupted metabolism and hormone regulation as well as an increased risk of various chronic diseases such as metabolic syndrome, diabetes, cardiovascular disease, and cancer^17^. In addition, it has been linked to immune deficiency, chronic inflammation, obesity, fatigue, and a higher likelihood of experiencing sleep disorder^18–21^. As a result, maintaining a healthy circadian rhythm is crucial for overall health and well-being, reducing the risk of adverse health effects and improving quality of life^22,23^. Considering the association between circadian rhythms and their impact on lifespan, along with the widespread adoption of recent technological advancements, we argue that smartwatches present a promising opportunity for leveraging digital biomarkers for longevity^24^. Smartwatches provide a practical means for continuously monitoring accelerometer data^25^ and heart rate data^26^, offering valuable insights into circadian rhythms.

Utilization of consumer smartwatches for data collection and analysis of potential digital biomarkers is, however, limited by a number of factors such as proprietary algorithms, limited data ownership, short lifespan, and variable wear time. Hence, ActiGraph devices, which are designed for research purposes, allow us to fully investigate the potential of future applications that could be implemented on these digital devices by using developed algorithms.

Furthermore, the application of machine learning (ML) to continuously collected data from wearables elucidates hidden patterns as digital phenotypes and facilitates subpopulation identification^27^. Conventional, expert-driven classification of disease or at-risk populations is limited by a lack of agreed ways of knowing the number of natural clusters in the populations of interest and determining the variables on which to base segmentation^28,29^. Instead, the use of a holistic and data-driven clustering approach has gained recognition as an alternative^29^. That is, each individual exists within multiple classes of health levels and provides various modalities of digitally measured physiological and behavioral data, which then correspond to multiple clusters of health status. Similar to those pioneered in the genomics fields, this method can result in advances in our understanding of the complex, multitude of components of disease etiology. To summarize, digital biomarkers and data-driven clustering approaches enable the use of precision medicine. These methods can classify a population into groups with unique characteristics or health risks and help individuals move from "unhealthy or at-risk" classes to "healthy" classes through intervention.

To date, the potential of using continuously collected data from wearables to explain longevity remains largely unstudied. In this study, we seek to investigate the use of 24-h activity profiles, such as accelerometer data, as a novel digital biomarker for longevity and tailored treatment. Our approach differs from previous research as it applies a data-driven approach to evaluate the association between 24-h accelerometer data and longevity measures in a nationally representative sample. This brings three distinct advantages in comparison to existing research. First, we apply population segmentation of wearable-based activity to the general U.S. adult population in the National Health and Nutrition Examination Survey (NHANES) cohort to increase the generalizability of our findings as compared to previously studying specific populations (i.e., chronic insomnia disorders^30^, middle-aged women^31^). Second, our ML-based clustering approach includes features depicting a detailed resolution of the 24-h activity profile, which represents comprehensive captures of both daily activity and physiological manifestations of the biological clock (e.g., ‘circadian rhythm’) such as the sleep/wake cycle^32^. In addition, the 24-h activity profile provides detailed information on an individual’s daily activity span, including timing and intensity, making it a richer source of information for health monitoring. Last, we examine the relationship between data-driven segmentation and different longevity outcomes that represent various dimensions of the current (i.e., inflammation and mortality) and predicted (i.e., biological age) health states of participants^10,33^.

## Results

### Baseline characteristics

Table 1 presents the baseline demographic and socioeconomic characteristics, medical history, and serum inflammatory biomarkers of the 7,297 study participants. In brief, the median age (interquartile range) was 51 (36–65) years, with 46.8% being male and 67.6% Non-Hispanic White. Common medical history included hypertension (49.0%), arthritis (27.7%), asthma (15.5%), and cancer (11.6%).

**Table 1:** Descriptive characteristics of 7,297 study participants.

Variables	Values
Age, median (interquartile range)	51 (36, 65)
Male, N (%)	3471 (46.8)
**Race/Ethnicity, N (%)**	
Mexican American	857 (8.2)
NH Black	1715 (11.1)
NH White	3000 (67.6)
Other	1019 (7.2)
Other Hispanic	706 (5.9)
Family income to poverty ratio, mean (SE)	2.5 (1.7)
Evern attended college, N (%)	4036 (63.0)
Married/Living with partner, N (%)	4210 (62.8)
**BMI groups, N (%)**	
Normal weight	2133 (28.5)
Overweight	2308 (33.3)
Obese	2782 (38.2)
**Employment status, N (%)**	
Working ≥ 40 h per week	2456 (39.1)
Working < 40 h per week	1211 (18.0)
Unemployed	3500 (40.9)
Unknown/Missing	130 (1.9)
**Household income, N (%)**	
< $20,000	1538 (14.5)
20,000–45,000	2046 (24.8)
45,000–75,000	1199 (19.0)
> 75,000	1857 (34.7)
Unknown/Missing	657 (7.0)
Current smoking, N (%)	1420 (19.3)
**Self-reported movement behaviors**	
Sleep, hours/day, mean (SE)	6.87 (1.4)
Sedentary behavior, hours/day, mean (SE)	6.58 (3.3)
Moderate intensity activity, hours/day, mean (SE)	0.92 (1.5)
Vigorous intensity activity, hours/day, mean (SE)	0.40 (1.1)
Sufficient MVPA, N (%)	4195 (60.2)
Having sleep trouble, N (%)	1915 (28.6)
Clinically diagnosed sleep disorder, N (%)	716 (10.1)
**Medical history, N (%)**	
Cardiovascular disease	497 (6.0)
Cancer	757 (11.6)
Stroke	312 (3.3)
Diabetes	1029 (10.7)
Hypertension	3785 (49.0)
Asthma	1114 (15.5)
Arthritis	2088 (27.7)
**Serum inflammatory biomarkers, mean (SE)**	
White blood cell count (10^9^ cells/L)	7.13 (2.19)
Neutrophils (10^9^ cells/L)	4.23 (1.66)
Lymphocytes (10^9^ cells/L)	2.11 (0.92)
NLR	2.22 (1.24)
SII	520.80 (328.0)
AISI	293.54 (245.6)

% and means (SE) are adjusted for weights. *N* number of subjects, *SE* standard errors, *NH* non-Hispanic, *BMI* body mass index, *MVPA* moderate- and vigorous-intensity physical activity, *NLR* neutrophil–lymphocyte ratio, *SII* systemic immune-inflammation index, *AISI* the aggregate index of systemic inflammation.

### Data-driven population segmentation and cluster profiling

**Figure 1:**
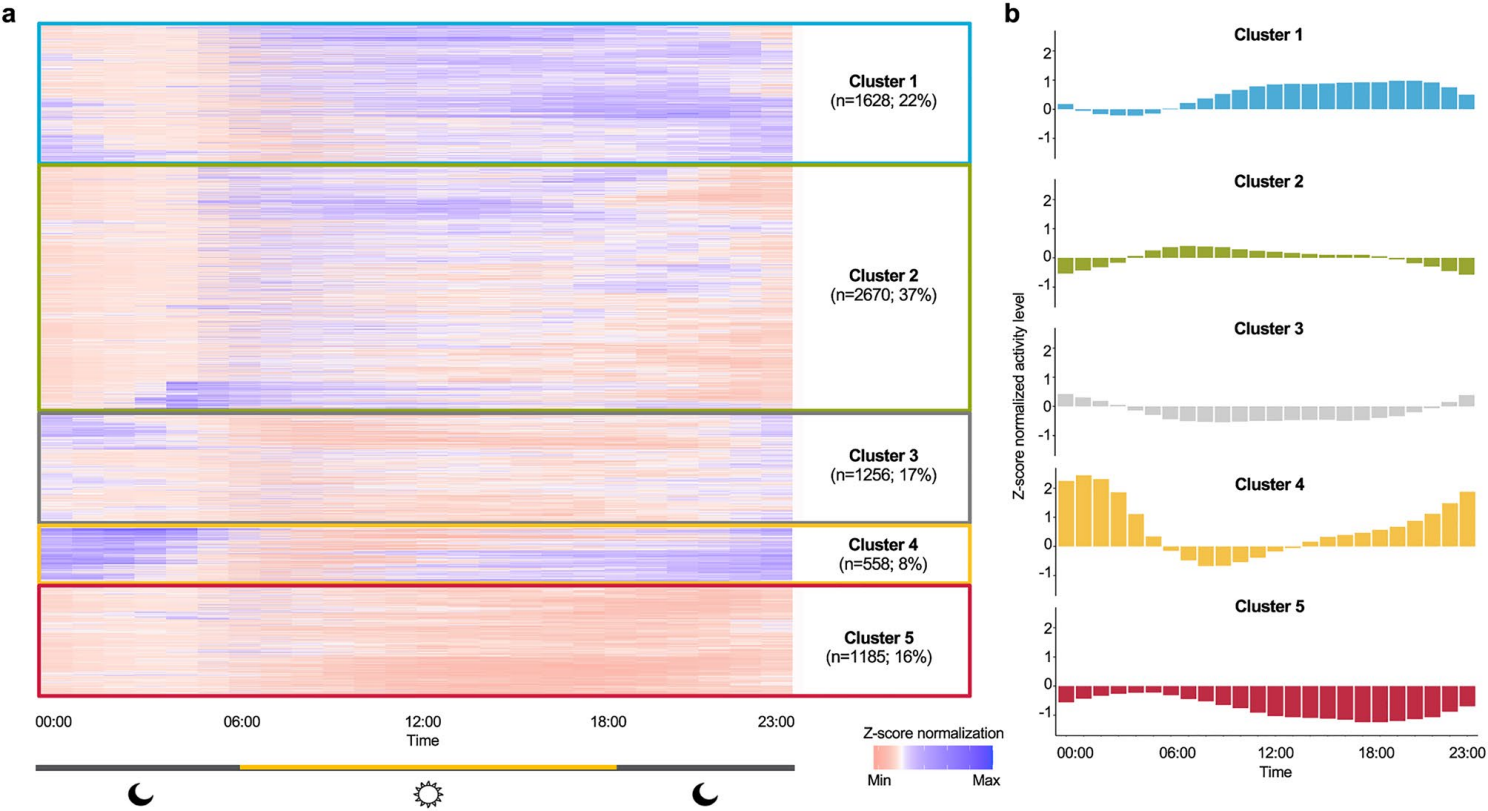
Cluster classification according to population segmentation of wearable-based accelerometer activity data. (**a**) Heatmap depicting the wearable-derived activity of 7,297 study participants over 24 h. (**b**) Graphical illustration of the hourly average accelerometer activity level by cluster. Values are Z-score normalized. Positive scores indicate activity levels above the population mean.

Applying hierarchical clustering to the wearable-derived hourly average activity data, we identified 22% (n = 1,628) participants in Cluster 1, 37% (n = 2,670) in Cluster 2, 17% (n = 1,256) in Cluster 3, 8% (n = 558) in Cluster 4, and 16% (n = 1,185) in Cluster 5. We observed distinct 24-h activity attributes by cluster (see Fig. 1). The rest/sleep hours for participants were defined based on the time period between 23:00 and 07:00, which is consistent with previous research on circadian rhythm and sleep, as well as their associations with various health outcomes^34^. Specifically, Cluster 1 showed a substantially higher activity level than the population average between 11:00 and 22:00 (Z-score: 0.75–0.98). During the rest/sleep period (i.e., 23:00–07:00), activity intensity is reduced dramatically and reached the nadir at 04:00. Cluster 2 participants showed above-average activity in the early morning between 05:00 and 10:00 (Z-score: 0.25–0.41), followed by the activity levels around the population mean during the daytime. We also observed a relatively earlier decline in accelerometer activity starting from 18:00. Cluster 3 exhibits low activity during active hours between 07:00–21:00 (Z-score: − 0.54 −  (− 0.06)) and increased activity above the population average between late night and early morning (i.e., 23:00–04:00, Z-score: 0.05–0.43). Cluster 4, the smallest cluster in size, is unique with its elevation of activity starting from 14:00 and high-level activity throughout the rest/sleep period, reaching its peak at 01:00 (Z-score: 2.44). Participants in Cluster 4 then had a substantial decline and dampened activity between 06:00 and 14:00, reaching the nadir at 08:00 (Z-score: − 0.67). Lastly, Cluster 5 has an all-time very low activity as shown in negative Z-scores. Particularly, daytime activity between 12:00 and 21:00 is significantly reduced (Z-score < − 1.0) in this cluster compared to the population mean.

We used the Student’s *t-*test for continuous variables and the Chi-square test for categorical variables to assess the statistical significance of demographic and socioeconomic characteristics, BMI groups, movement behaviors, sleep quality, and medical history by clusters. In data-driven clusters, all variables except asthma were statistically significant (see Table 2). Clusters 1 and 4 were on average young adults (median ages 41 and 36). Clusters 2 and 3 included middle-aged adults (median ages 53 and 51). Cluster 5 consisted of an older population aged between 60 and 80 years. Comparing two middle-aged clusters, Cluster 3 had significantly higher percentages of NH Black (18% vs. 8%) and obesity (44% vs. 36%), higher unemployment rates (52% vs. 33%), fewer participants working ≥ 40 h/week (32% vs. 47%), and lower household income than Cluster 2. In addition, Cluster 3 participants reported spending more time in sedentary behaviors and less time in moderate- or vigorous-intensity activities, with a lower proportion (55%) meeting recommended moderate- and vigorous-intensity physical activity (MVPA) guidelines, in contrast to Cluster 2 which had a higher percentage (65%) meeting the guidelines. Cluster 3 had a greater proportion of participants reporting sleep disturbances and clinically diagnosed sleep disorders, as well as a higher prevalence of cardiovascular disease, cancer, stroke, diabetes, hypertension, and arthritis. When comparing the two young adult clusters, Cluster 4 had a larger percentage of males (55% vs. 36%), being non-Hispanic Black (20% vs. 11%) and unmarried (55% vs. 36%), and obesity (40% vs. 31%), having lower income levels and family income to poverty ratio than Cluster 1. Unlike middle-aged clusters, we observed no significant differences in the distributions of working ≥ 40 h/week (~ 40%), working < 40 h/week (~ 20%), and unemployed (~ 30%) between these two groups. In addition, there were no significant differences in the prevalence of medical conditions. In the comparison of movement behaviors, participants in Cluster 4 demonstrated a bimodal relationship, with longer periods of both sedentary and moderate- and vigorous-intensity activity durations compared to those in Cluster 1. Furthermore, our analysis revealed five distinctive characteristics of Cluster 4, which included the highest percentages of NH Black and current smokers, the lowest family income to poverty ratio, the shortest sleep duration, and the longest MVPA duration. Finally, Cluster 5, the eldest population, had the highest number of medical conditions and reported the longest sleep and sedentary time.

**Table 2:** Cluster characteristics.

Variables	Cluster 1 (n = 1628; 22%)	Cluster 2 (n = 2670; 37%)	Cluster 3 (n = 1256; 17%)	Cluster 4 (n = 558; 8%)	Cluster 5 (n = 1185; 16%)	*p*-value
**Characterization**	High activity	Low activity	Mild CR disruption	Severe CR disruption	Very low activity	
Age, median (interquartile range)	41 (31, 53)	53 (40, 64)	51 (34, 64)	36 (26, 50)	71 (60, 80)	< 0.001
Male, N (%)	583 (35.6)	1244 (47.2)	650 (50.4)	303 (55.1)	691 (55.8)	< 0.001
**Race/Ethnicity, N (%)**						< 0.001
Mexican American	271 (12.9)	366 (8.0)	79 (5.2)	58 (10.2)	83 (3.6)	
NH Black	333 (10.5)	503 (8.1)	410 (17.7)	185 (20.4)	284 (9.9)	
NH White	560 (60.0)	1176 (72.6)	430 (61.2)	169 (51.3)	665 (79.5)	
Other	246 (7.2)	373 (6.7)	220 (10.2)	82 (8.5)	98 (4.6)	
Other Hispanic	218 (9.4)	252 (4.6)	117 (5.8)	64 (9.5)	55 (2.5)	
Family income to poverty ratio, mean (SE)	2.33 (1.68)	2.78 (1.60)	2.38 (1.71)	1.93 (1.56)	2.32 (1.59)	< 0.001
Ever attended college, N (%)	874 (60.7)	1519 (65.4)	792 (68.7)	286 (57.0)	565 (57.0)	< 0.001
Married/Living with partner, N (%)	976 (63.9)	1740 (70.5)	630 (52.9)	245 (44.9)	619 (56.9)	< 0.001
**BMI groups, N (%)**						< 0.001
Normal weight	558 (34.4)	779 (28.4)	350 (25.9)	182 (33.1)	264 (20.2)	
Overweight	540 (34.7)	905 (35.7)	348 (30.0)	160 (26.7)	355 (30.5)	
Obese	526 (31.0)	971 (35.9)	540 (44.1)	213 (40.2)	532 (49.3)	
**Employment status, N (%)**						< 0.001
Working ≥ 40 h per week	641 (42.5)	1115 (47.2)	346 (32.2)	219 (41.5)	135 (16.8)	
Working < 40 h per week	390 (24.1)	444 (18.3)	167 (14.6)	120 (23.2)	90 (8.8)	
Unemployed	557 (30.1)	1064 (32.8)	723 (51.9)	206 (33.0)	950 (73.5)	
Unknown/Missing	40 (3.3)	47 (1.7)	20 (1.3)	13 (2.3)	10 (0.9)	
**Household income, N (%)**						< 0.001
< $20,000	308 (14.3)	422 (9.5)	308 (18.5)	152 (23.0)	348 (21.0)	
20,000–45,000	475 (24.7)	683 (20.9)	356 (26.9)	170 (31.2)	362 (30.7)	
45,000–75,000	280 (20.2)	468 (20.0)	189 (17.1)	85 (16.2)	177 (17.6)	
> 75,000	395 (32.6)	851 (43.0)	307 (31.6)	95 (21.8)	209 (23.9)	
Unknown/Missing	170 (8.1)	246 (6.6)	96 (5.8)	56 (7.9)	89 (6.8)	
Current smoking, N (%)	320 (20.7)	413 (15.0)	309 (24.4)	184 (32.9)	194 (18.3)	< 0.001
**Self-reported movement behaviors**						
Sleep, hours/day, mean (SE)	6.83 (1.23)	6.93 (1.23)	6.70 (1.42)	6.47 (1.59)	7.15 (1.58)	< 0.001
Sedentary behavior, hours/day, mean (SE)	5.86 (3.22)	6.31 (3.18)	7.20 (3.51)	6.13 (3.47)	7.59 (3.26)	< 0.001
Moderate intensity activity, hours/day, mean (SE)	1.07 (1.56)	0.97 (1.53)	0.78 (1.33)	1.48 (2.05)	0.49 (1.05)	< 0.001
Vigorous intensity activity, hours/day, mean (SE)	0.48 (1.20)	0.43 (1.00)	0.30 (0.89)	0.71 (1.62)	0.15 (0.66)	< 0.001
Sufficient MVPA, N (%)	1068 (67.2)	1646 (64.5)	659 (54.8)	384 (71.1)	438 (37.8)	< 0.001
Having sleep trouble, N (%)	391 (27.2)	654 (26.9)	379 (32.6)	126 (23.0)	365 (34.0)	0.039
Clinically diagnosed sleep disorder, N (%)	100 (6.9)	231 (9.1)	160 (13.6)	53 (7.1)	172 (15.4)	< 0.001
**Medical history, N (%)**						
Cardiovascular disease	36 (2.0)	129 (4.5)	94 (7.7)	9 (1.3)	229 (16.7)	< 0.001
Cancer	73 (5.2)	265 (10.9)	136 (13.5)	26 (5.5)	257 (24.3)	< 0.001
Stroke	19 (1.1)	68 (2.0)	50 (4.1)	14 (2.1)	161 (10.4)	< 0.001
Diabetes	95 (4.0)	295 (7.7)	243 (17.4)	35 (4.2)	361 (25.9)	< 0.001
Hypertension	557 (33.1)	1428 (50.5)	669 (51.3)	212 (32.8)	919 (73.6)	< 0.001
Asthma	241 (16.2)	364 (14.2)	231 (17.7)	102 (17.9)	176 (14.8)	0.139
Arthritis	282 (18.0)	754 (27.6)	412 (31.4)	89 (13.9)	551 (45.8)	< 0.001
**Serum inflammatory biomarkers, mean (SE)**						
White blood cell count (10^9^ cells/L)	6.98 (2.11)	7.04 (2.01)	7.10 (2.13)	7.34 (2.16)	7.47 (2.75)	< 0.001
Neutrophils (10^9^ cells/L)	4.09 (1.64)	4.19 (1.59)	4.16 (1.67)	4.26 (1.78)	4.52 (1.78)	< 0.001
Lymphocytes (10^9^ cells/L)	2.12 (0.64)	2.08 (0.67)	2.14 (0.80)	2.28 (0.74)	2.08 (1.71)	0.002
NLR	2.03 (0.88)	2.20 (1.18)	2.18 (1.38)	2.01 (1.11)	2.65 (1.63)	< 0.001
SII	487.85 (243.77)	520.29 (324.54)	520.12 (342.60)	487.84 (264.53)	584.28 (434.28)	< 0.001
AISI	271.46 (208.53)	285.47 (233.88)	294.74 (260.16)	271.12 (181.78)	352.15 (318.81)	< 0.001

% and means (SE) are adjusted for weights. *N* number of subjects, *CR* circadian rhythm, *SE* standard errors, *NH* non-Hispanic, *BMI* body mass index, *MVPA* moderate- and vigorous-intensity physical activity, *NLR* neutrophil–lymphocyte ratio, *SII* systemic immune-inflammation index, *AISI* the aggregate index of systemic inflammation.

### Differences in inflammatory biomarkers, biological age, and mortality according to cluster classification

**Figure 2:**
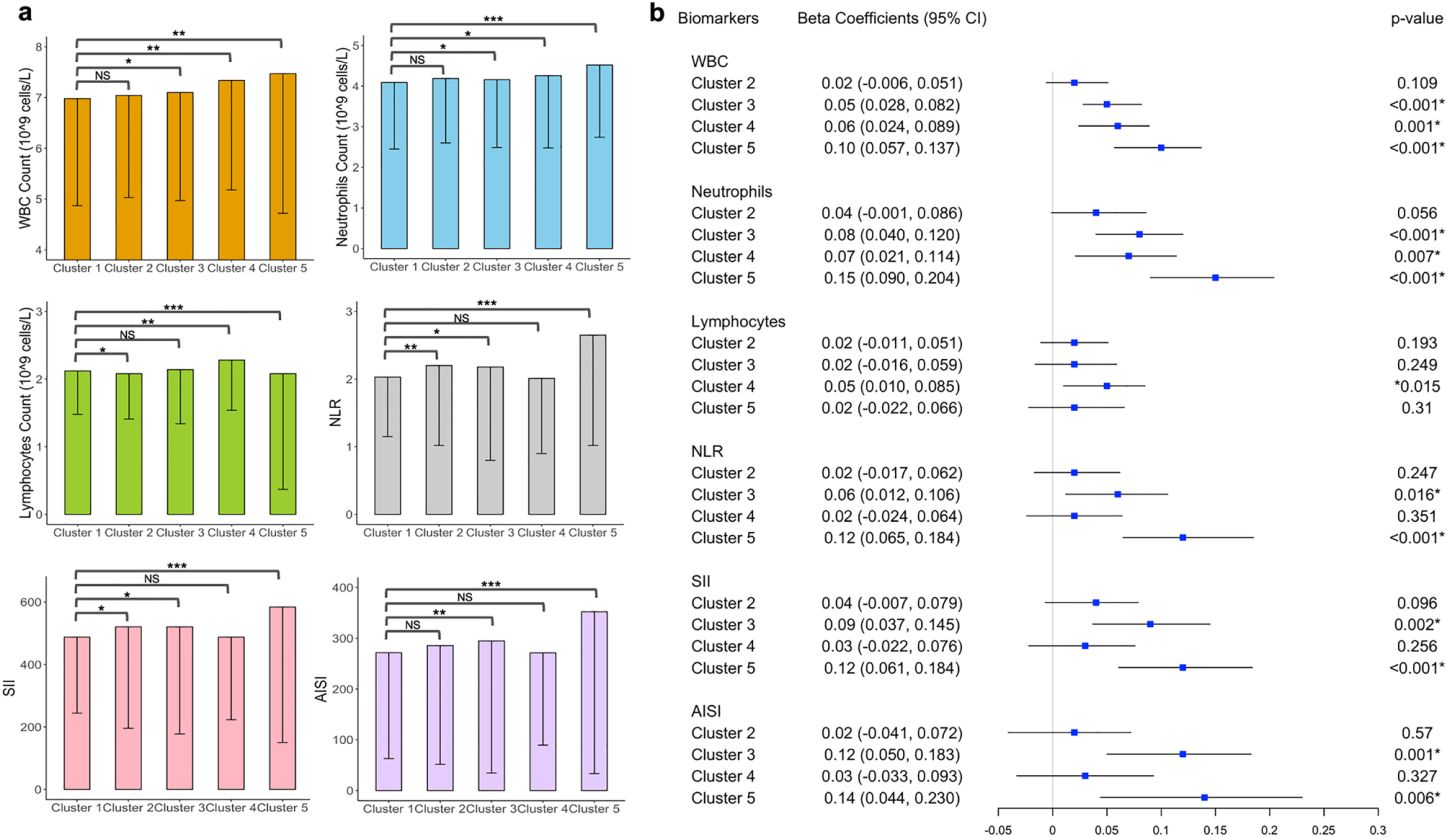
Associations of clusters with white blood-cell-based inflammatory markers. (**a**) Comparisons of clusters (mean ± SE) on WBC count, neutrophils count, lymphocyte count, NLR, SII, and AISI, respectively. Statistical significance is set at *p *< 0.05 (*), < 0.01 (**), < 0.001 (***), and *p *> 0.05 = not significant (NS). A survey-weighted generalized linear model was used. (**b**) Forest plot of beta coefficients and 95% confidence intervals (CI). Cluster 1 is a reference. The model is adjusted for age, sex, race/ethnicity, and employment status. Statistical significance is set at *p *< 0.05 (*). All *p*-values were calculated using log-transformed values of outcomes. *WBC* white blood cell, *NLR* neutrophil–lymphocyte ratio, *SII* systemic immune-inflammation index, *AISI* the aggregate index of systemic inflammation. *SE* standard errors.

**Figure 3:**
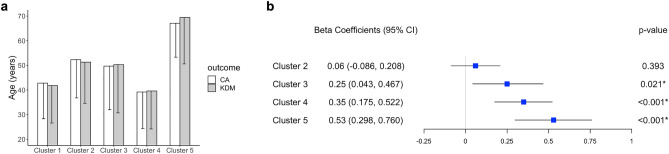
Associations of clusters with KDM biological age. (**a**) Comparison of clusters (mean ± SE) on the chronological age (CA) and Klemera-Doubal method (KDM) biological age. (**b**) Forest plot of beta coefficients and 95% confidence intervals (CI). Cluster 1 is a reference. The model is adjusted for age, sex, race/ethnicity, and employment status. Statistical significance is set at *p *< 0.05 (*). All *p*-values were calculated using log-transformed values of outcomes. *SE* standard errors.

**Figure 4:**
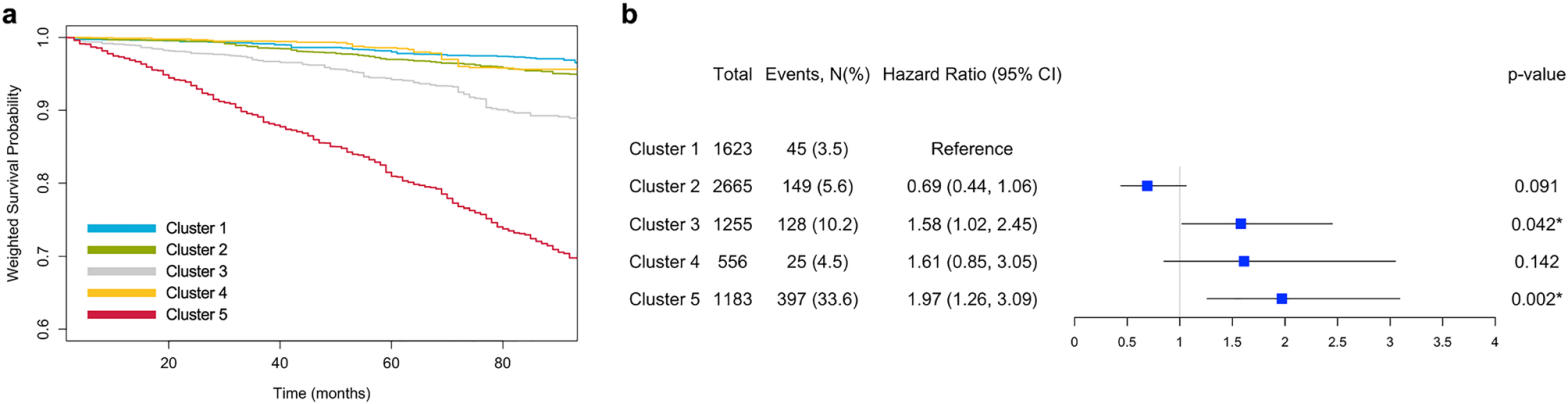
Associations of clusters with all-cause mortality. (**a**) Weighted Kaplan–Meier curve of time to all-cause mortality by cluster. (**b**) Forest plot of hazard ratios of all-cause mortality and 95% confidence intervals (CI). Cluster 1 is a reference. We used a survey-weighted Cox proportional hazard model adjusted for age, sex, race/ethnicity, and employment status. Statistical significance is set at *p *< 0.05 (*).

We assessed the associations between data-driven clusters and white blood cell-based inflammatory biomarker levels (see Fig. 2), Klemera-Doubal (KDM) biological age (see Fig. 3), and all-cause mortality (see Fig. 4). Across health-related outcomes, we observed Cluster 1 to perform best and Cluster 5 to perform worst. These associations hold even after adjusting for covariates.

Specifically, Clusters 3, 4, and 5 had 0.05–0.10 log-unit higher white blood cell counts and 0.08–0.15 log-unit higher neutrophil counts compared to Cluster 1 (see Fig. 2). In addition, Cluster 4 was associated with 0.05 log-unit higher (95% CI: 0.010–0.085) lymphocyte count. Clusters 3 and 5 were associated with 0.06–0.12 and 0.09–0.14 log-unit increases in NLR and hematological aggregate indices for systemic inflammation expressed in SII and AISI (all *p *< 0.05).

For KDM biological age, we noticed an accelerated advance of the biological aging process in Clusters 3 to 4 to 5 (See Fig. 3). Specifically, participants in Cluster 3 had a biological age advance of 0.25 log-years (equivalent to 1.28 years, 95% CI: 0.043–0.467) greater than those in Cluster 1. Participants in Clusters 4 and 5 exhibited an even faster rate of biological age advance, at 0.35 log-years (equivalent to 1.42 years, 95% CI: 0.175–0.522) and 0.53 log-years (equivalent to 1.70 years, 95% CI: 0.298–0.760), respectively.

Finally, we analyzed all-cause mortality risk associated with the clusters (see Fig. 4). Cluster 3 was associated with 1.58 (95% CI: 1.02–2.45) times higher, and Cluster 5 was associated with 1.97 (95% CI: 1.26–3.09) times higher all-cause mortality risks than Cluster 1. Although statistical significance was not reached, we also found a similar trend of increased mortality risks in Cluster 4 (HR 1.61, 95% CI: 0.85–3.05).

## Discussion

We applied a data-driven clustering approach to identify population segments based on 24-h accelerometer activity data collected using a wearable device in U.S. adults. Based on the 24-h activity profiles, we found five distinct clusters, which we describe as follows. Cluster 1 represents a “High activity” group maintaining elevated levels of activity throughout the day. Cluster 2 portrays a “Low activity” group, exhibiting a diurnal pattern similar to that of Cluster 1 but with lower overall activity levels throughout the day and a faster decline from early evening. Clusters 3 and 4 represent the “Mild circadian rhythm (CR) disruption” group and the “Severe CR disruption” group, respectively. Cluster 3 participants exhibit increased nocturnal activity between 23:00 and 04:00, while their daytime activity remains low. Cluster 4 is characterized by extremely low activity from morning to early afternoon, a gradual elevation in the evening, notably high activity during rest/sleep hours, and a sharp fall in the morning. These activity patterns are indicative of circadian misalignment or disrupted rhythm, as they do not align well with normal light-darkness schedules. Therefore, we have classified these clusters as having circadian rhythm disruption. Lastly, Cluster 5 represents “Very low activity” group.

**Figure 5:**
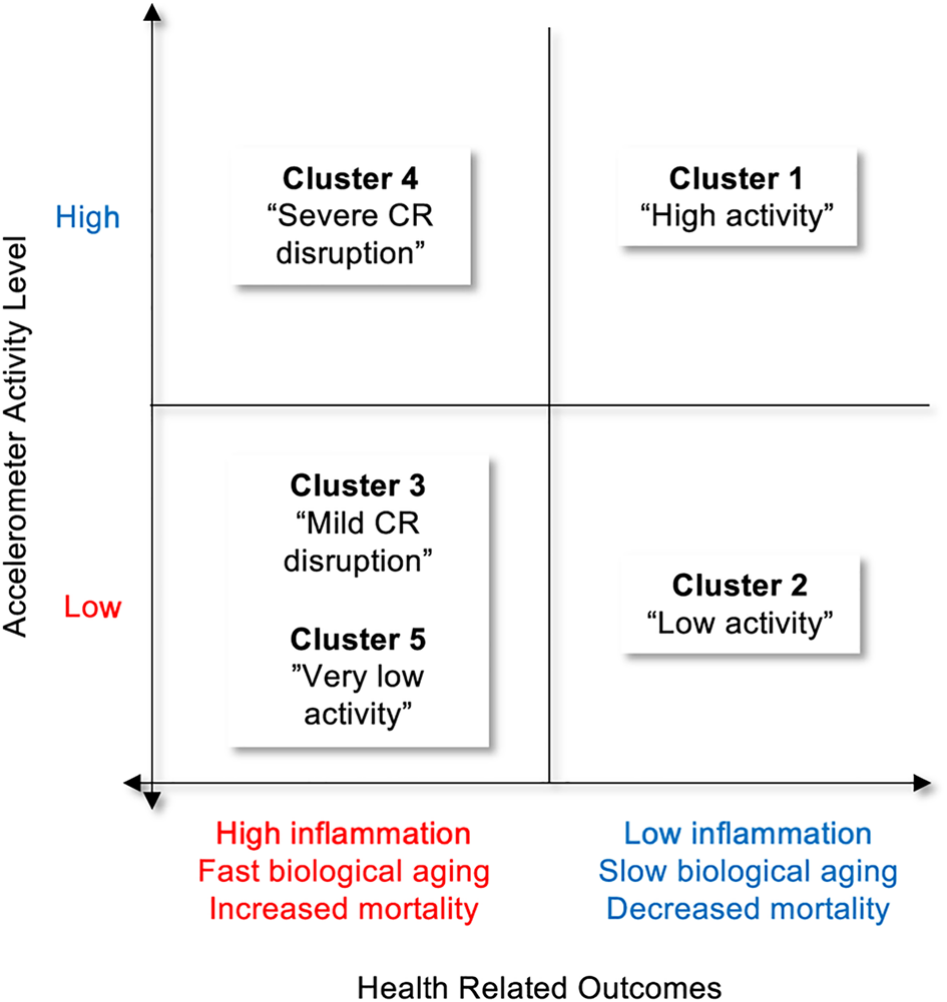
Five clusters in relation to accelerometer activity level and health-related outcomes. *CR* circadian rhythm.

We demonstrated that clusters are significantly associated with baseline characteristics, as determined by *t-*test and Chi-square tests. The identified clusters are clearly differentiated by demographic and socioeconomic factors, movement behaviors, and medical conditions. Furthermore, our generalized linear models and Cox proportional hazards models revealed significant associations and gradient effects between cluster membership and three longevity outcomes, namely inflammatory biomarker levels, biological age, and all-cause mortality. Across all health-related outcomes, “High activity” group (Cluster 1) tends to have the best performance, with the lowest values of inflammation levels, biological age advance, and mortality. This was followed by “Low activity” (Cluster 2), “Mild CR disruption” (Cluster 3), and “Severe CR disruption” (Cluster 4). “Very low activity” (Cluster 5) group performed worst, with the highest inflammation levels, mortality risk, and biological age (see Fig. 5).

There were, however, a few exceptions. “Severe CR disruption”, consisting of young adults aged 30–40 years, was significantly associated with increased inflammatory biomarkers and accelerated biological age, but not with all-cause mortality and medical histories. This finding suggests that young adults with circadian misalignment may seem ostensibly healthy because they have no apparent signs of medical conditions and show high levels of activity, but in fact, are undergoing health deterioration and unhealthy aging. In middle-aged adults, having some degree of circadian cycle disturbance together with a low activity level (“Mild CR disruption”) resulted in substantially higher inflammatory biomarker levels, mortality risk, and biological age compared to having low activity alone. This highlights the growing importance of circadian alignment in older populations to achieve healthy longevity.

Unlike physical activity or nutrition, there is still a lack of understanding regarding how to utilize or correct biological timing for health benefits. Current public health interventions are largely focusing on increasing physical activity levels or eating healthy, with less attention on targeting the circadian clock. Mounting evidence indicates that circadian disruption has significant consequences for various health outcomes, including performance, well-being, physical and mental health, and longevity^24,35^. As such, smartwatches and wearables offer a timely, unobstructed, and convenient method for monitoring and assessing circadian rhythms. With the increasing uptake of digital devices, circadian clock-based therapeutics have enormous potential for maximizing health benefits and promoting healthy aging at individual and population levels^36–39^. Coupled with machine learning algorithms, digitization of such passive behavior data has an unrecognized potential as novel digital biomarkers for longevity and advancing personalized interventions, automated health event prediction, and population-level prevention. As an implication of this study, we can utilize wearable data as a digital biomarker and deliver personalized intervention via digital devices to successfully promote synchronization with the diurnal cycle, i.e., migrate “unhealthy or at-risk” individuals to “healthy” clusters. Young adults with an impaired circadian cycle, for example, may be given recommendations such as timely light exposure, exercise at specific times, melatonin ingestion, or utilizing digital technology for monitoring to improve their sleep–wake cycle^40,41^. Meanwhile, older adults with low activity levels may be recommended to increase their physical activity and engage in other healthy behaviors to reduce the risk of age-related diseases and increase strength and mobility.

There are potential limitations of this study. First, the validity of the feature selection must be verified on new data, unseen from the model during the development phase. Second, this is a retrospective analysis and cannot establish causal relationships between the observed associations. Third, we use only 7-day accelerometer data, and a longer duration of monitoring would provide a more precise and accurate classification of clusters. Fourth, unmeasured environmental factors or residual confounding could have affected accelerometry measurements. Similarly, non-wear time and missing accelerometry measures may influence the activity output. However, the impact is minimal as we selected participants with complete 5-min epoch information in the analysis. Next, data on shift work status and work schedule are missing, and it is possible that the clusters we identified may be biased toward including shift workers and therefore not representative of the general population with normal work schedules. However, we believe that the impact of shift work status would not fully explain our findings for two reasons. First, we found that the employment status in our data did not significantly differ comparing the group with the severe circadian disruption to the group without disruption. Second, controlling for employment status did not affect the original associations in the generalized linear models and Cox proportional hazards models. Lastly, the initial cost of purchasing a wearable device may not appear to be cost-effective from a population perspective in the short term ($250.00 per unit). However, it could potentially become cost-effective in the long term due to the following reasons: (1) the widespread use of smartphones and smartwatches makes them scalable solutions for continuous data collection in a large population; (2) wearables are more economical in the long run when compared to traditional methods such as clinical visits or lab tests, which require physical encounters and can incur costs for each visit; (3) as technology advances, the availability of low-cost wearable devices and commercial smartwatches with accelerometer functionality is increasing.

Nevertheless, this study offers the following contributions over previous research. This study used wearable-based accelerometer activity data to segment a nationally representative sample of the U.S. population. A novel, detailed resolution of the 24-h activity profile elucidates distinct cluster profiles and highlights circadian misalignment and rhythm disruption to play a critical role in longevity measures of inflammation, biological age, and mortality. With this work, we add a meaningful contribution to current research in the field demonstrating the potential for the digitization of human longevity measures based on continuous wearable-based activity data. A digital biomarker for longevity has enormous potential for digital phenotyping, personalized intervention, population-level prevention, and remote monitoring of people’s health. It is also a critical step toward achieving the aim of precision medicine. Future studies with prospective and repeated assessments using digital devices are warranted.

## Methods

### Participants

**Figure 6:**
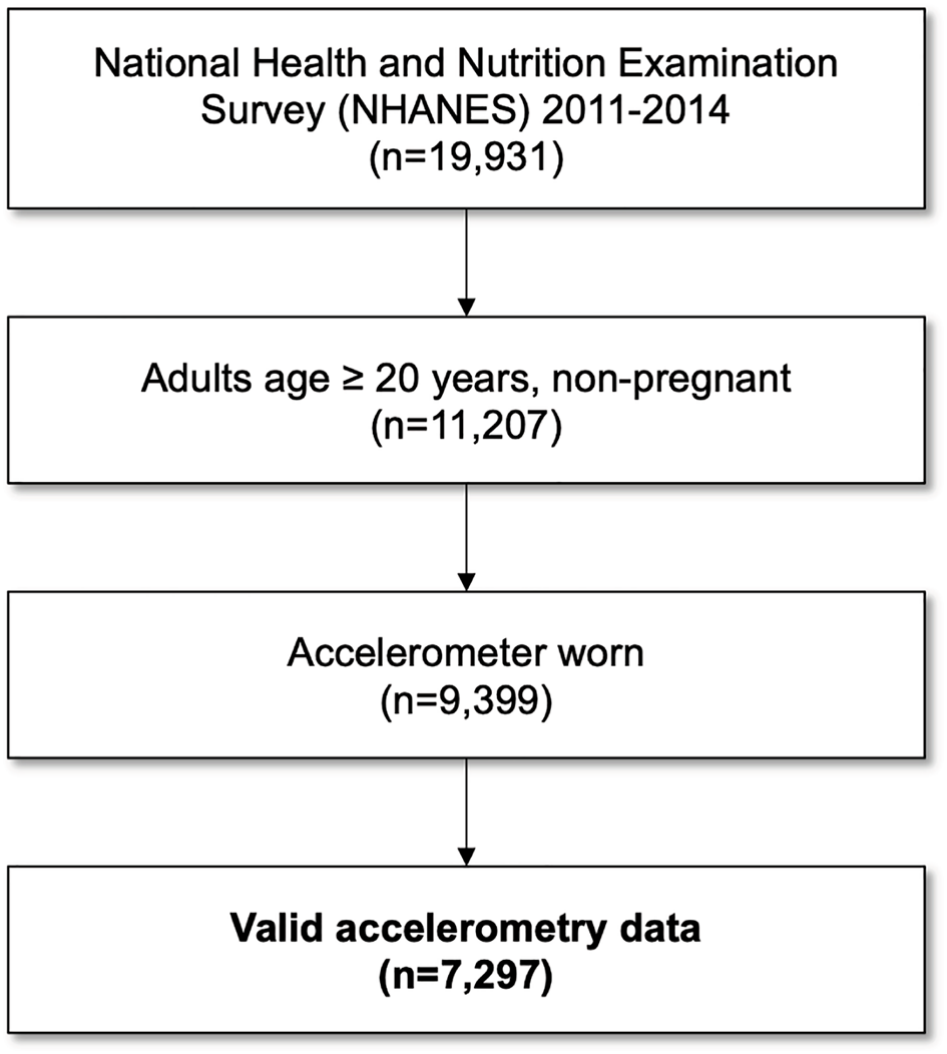
Flowchart for inclusion of study participants.

We utilized data from the NHANES, a nationwide cross-sectional survey conducted by the Centers for Disease Control and Prevention to assess the health and nutritional status of adults and children in the United States^42^. The NHANES applies a stratified, multistage probability sampling design to generate a weighted, representative sample of the U.S. population. The National Center for Health Statistics Ethics Review Board approved the NHANES study protocols (NCHS IRB/ERB Protocol Number: #2011–17), and all participants provided written informed consent. All methods were performed in accordance with the Declaration of Helsinki. For the present study, we selected non-pregnant adults ≥ 20 years who had validated accelerometer recordings from NHANES 2011–2014 cycles, for which 24-h accelerometer data were available. Participants had valid accelerometer data if satisfying a minimum of 16 h of daily wearing time for 4 or more days. In addition, participants’ accelerometer data should be recorded in a continuous and time-series manner, without missing 5-min epochs over 24 h. The study included 7,297 participants in the analysis (see Fig. 6).

### Serum inflammatory biomarker measures

Blood sample collection, laboratory methods, and detailed processing instructions are described in the NHANES Laboratory/Medical Technologists Procedure Manual^43^. The blood analyzer provided white blood cell count, neutrophil count, lymphocyte count, and neutrophil-to-lymphocyte ratio (NLR). We additionally computed two hematological indexes for systemic inflammation, the systemic immune-inflammation index (SII) and the aggregate index of systemic inflammation (AISI), using the following formulae^44,45^:

- SII = neutrophil x platelet/lymphocyte count
- AISI = neutrophil x monocyte x platelet/lymphocyte count.

### Biological aging measure

We used the modified Klemera–Doubal method (KDM) for biological age prediction^10,46^. We chose KDM biological age as it has shown to be more accurate than other alternatives for the prediction of morbidity, mortality, and indicators of health span^47,48^. We included 11 biomarkers in the biological age estimation using BioAge R package 0.1.0.^49^: albumin, alkaline phosphatase, total cholesterol, creatinine, HbA1c, systolic blood pressure, blood urea nitrogen, uric acid, lymphocyte percentage, mean cell volume, and white blood cell count.

### Mortality data

We used a publicly available file from the National Centre for Health Statistics (NCHS) with certified death records from the National Death Index (NDI). Follow-up periods are from the date of the interview to the registered date of death for the deceased or the end of the follow-up period (December 31, 2015) for those who survived.

### Data processing of wearable-derived accelerometer activity data

All participants aged 6 years and older during the 2011–2012 cycle and all participants aged 3 years and older during the 2013–2014 cycle wore an ActiGraph GT3X + (Actigraph, Pensacola, FL) accelerometer on the non-dominant wrist for 7 consecutive 24-h periods. The wearable collected raw signals on the x, y, and z axes with a sampling rate of 80 Hz. NHANES processed, flagged, and summarized accelerometer data at the minute level in Monitor-Independent Movement Summary (MIMS) units, which is a non-proprietary, open-source, device-independent universal summary metric . We applied a series of quality control and data processing steps to identify valid accelerometer data suitable for our analysis. First, we included accelerometer data from participants who wear the accelerometer for 16 h or more per day for at least 4 days, not including the first day of wear, which was excluded from data processing. Previous research indicates that for population-level analyses 16 h of wear time for 4 or more days were sufficient to generate stable group-level estimates of activity using accelerometer data^51^. The wear time was determined through wake-wear, sleep-wear, non-wear, and unknown estimates calculated based on a machine learning algorithm^52^. Second, we further identified accelerometer data in completed 5-min epochs per day (i.e., non-missing 288-time slices) in order to capture continuous time series of activity levels over the course of 24 h. The rationale of this step is to identify potential non-continuity and disruption in data that cannot be assessed with the first criterium. Participants with sufficient valid wear time may still display successive missing values for a prolonged period, which would generate incomplete 24-h activity profiles and impact our analyses. Finally, in accordance with previous studies, we set MIMS triaxial values as missing for the following three conditions: (1) the MIMS triaxial value is coded as ‘− 0.01’ (variable name: PAXMTSM); (2) estimated wake/sleep/wear status during the minute is “non-wear” (variable name: PAXPREDM); (3) minute data quality flag count is larger than ‘0’ (variable name: PAXQFM)^51,53^.

### Feature selection and hierarchical clustering

For participants with valid accelerometer data, we used their MIMS triaxial values across all available days (i.e., the sum of MIMS x-, y-, and z-axis values) from the minute-level summary file (file name: PAXMIN; variable name: PAXMTSM) to calculate the hourly activity levels over 24 h. This results in a vector with 24 entries per participant, of which each entity represents the hourly average activity level of the given hour, expressed from 00:00 (1^st^ entity) to 23:00 (24^th^ entity). Previous studies have shown that hourly variation of the activity assessed over 24 h using accelerometers provides meaningful information about the general adult population^54,55^. We then applied recursive feature elimination to identify an optimal set of features from the aforementioned 24 input entities of activity levels that significantly separate clusters in our data (see Supplementary Fig. 1). Using only the 16 selected features, we applied a hierarchical clustering approach using Ward’s linkage algorithm with Euclidean distances for population segmentation of wearable-based accelerometer activity data in U.S. adults (see Supplementary Fig. 1). All analysis was performed using R software 4.1.2 and RStudio 2022.07.1. In particular, we used the caret package 6.0–90 for feature selection. R packages cluster 2.1.2, mclust 5.4.10, dendextend 1.16.0, ggdendro 0.1.23, and factoextra 1.0.7 were implemented for hierarchical clustering algorithms and result visualizations.

### Covariates

We obtained additional information on characteristics a priori that would be associated with inflammatory biomarkers, biological age and mortality based on previous research^36,56,57^: Age, sex, race/ethnicity, family income to poverty ratio, education, marital status, employment status, household income, smoking status, sleep hours and quality, and history of cardiovascular disease, cancer, stroke, diabetes, hypertension, asthma, and arthritis. We calculated the body mass index (BMI) by dividing weight in kilograms by height in meters squared. BMI was further categorized into three groups: Normal weight (BMI < 25), Overweight (BMI 25–29.9), and Obese (BMI ≥ 30). Durations of different movement behaviors such as sleep, sedentary, moderate-intensity, and vigorous-intensity physical activity durations were assessed by self-report. We categorized participants to have sufficient moderate- and vigorous-intensity physical activity (MVPA) if he/she meets guidelines recommended by the Physical Activity Guidelines for Americans (i.e., 150 min or more moderate-intensity activity per week or 75 min or more vigorous-intensity activity per week)^58^.

### Statistical analysis

To account for the complex survey design and produce representative estimates of the U.S. population, we applied four-year survey weights to all statistical procedures using the survey package 4.1–1 to adjust for unequal selection probability and non-response bias in accordance with NHANES analytical guidelines^59^. In descriptive statistics, we obtained the population means, proportion, and standard errors (SE) with the entire sample (see Table 1) and by cluster (see Table 2). We conducted Student’s *t-*test or Chi-square tests for continuous or categorical variables to compare baseline characteristics by cluster.

For associations of clusters with serum inflammatory biomarkers (i.e., white blood cell count, neutrophil count, lymphocyte count, NLR, SII, and AISI) and the Klemera–Doubal method-based biological age, we used the survey-weighted generalized linear models with and without adjusting covariates (see Figs. 2 and 3). Considering the skewed distribution, dependent variables were log-transformed in these models. In addition, we depicted the differences in all-cause mortality based on clusters in a weighted Kaplan–Meier curve with R package adjustedCurves 0.9.1 (see Fig. 4a). We further fitted a survey-weighted Cox proportional hazard model adjusting for covariates to estimate HRs and 95% CI for associations between clusters and all-cause mortality (see Fig. 4b). The proportional hazard assumption was satisfied. Based on a backward selection, we included age, sex, race/ethnicity, and employment status in adjusted models. We conducted sensitivity analyses to check the interactions between clusters and covariates, and no effect modification was observed. Statistical significance was at two-sided *p *< 0.05.

## Supplementary Information

Supplementary Information.

## Data Availability

The NHANES data that support the findings of this study are available from CDC Centers for Disease Control and Prevention website [https://wwwn.cdc.gov/nchs/nhanes/Default.aspx].
