# Supplementary material for: Wearable-based accelerometer activity profile as digital biomarker of inflammation, biological age, and mortality using hierarchical clustering analysis in NHANES 2011–2014
Source: Sci Rep. 2023 Jun 8;13:9326. doi: 10.1038/s41598-023-36062-y (PMC10250365; doi:10.1038/s41598-023-36062-y)
Supplement: Supplementary file 1 — Supplementary Information. [file 41598_2023_36062_MOESM1_ESM.docx]

**Title:** Wearable-based Accelerometer Activity Profile as Digital Biomarker of Inflammation, Biological Age, and Mortality Using Hierarchical Clustering Analysis in NHANES 2011-2014

**Authors:** Jinjoo Shim, MSc^1*^, Elgar Fleisch, PhD^1,2^, Filipe Barata, PhD^1^

**
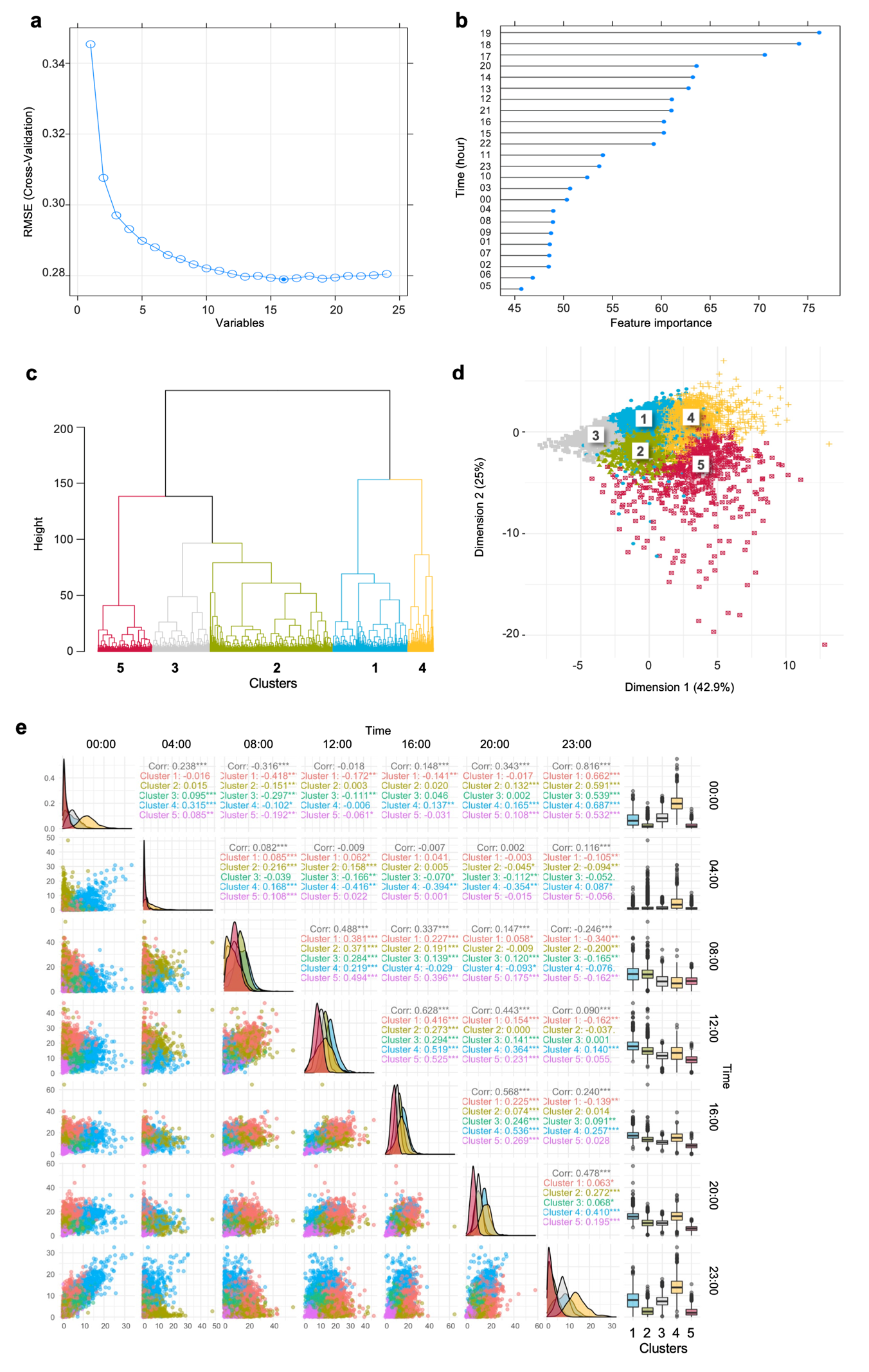
**

**Supplementary Fig. 1 Development of ML model for wearable-based population segmentation.** **a** Feature selection using recursive feature elimination. **b** Random forest feature importance plot. **c-d** Dendrogram and scatter plot of clusters depicting their hierarchical composition based on 16 selected features. **e** Distribution of observed accelerometer activity at different time points in a 24-hour span by clusters. *RMSE* root mean squared error.
